# A new mild hyperthermia device to treat vascular involvement in cancer surgery

**DOI:** 10.1038/s41598-017-10508-6

**Published:** 2017-09-12

**Authors:** Matthew J. Ware, Lam P. Nguyen, Justin J. Law, Martyna Krzykawska-Serda, Kimberly M. Taylor, Hop S. Tran Cao, Andrew O. Anderson, Merlyn Pulikkathara, Jared M. Newton, Jason C. Ho, Rosa Hwang, Kimal Rajapakshe, Cristian Coarfa, Shixia Huang, Dean Edwards, Steven A. Curley, Stuart J. Corr

**Affiliations:** 1 Department of Surgery, Baylor College of Medicine, Houston, TX 77030 USA; 2 Faculty of Biochemistry, Biophysics and Biotechnology, Jagiellonian University, Gronostajowa 7 St., Kraków, 30-387 Poland; 3 Department of Otolaryngology-Head and Neck Surgery, Baylor College of Medicine, Houston, TX 77030 USA; 4 Interdepartmental program in Translational Biology and Molecular Medicine, Baylor College of Medicine, Houston, TX 77030 USA; 5 Department of Surgical oncology, MD Anderson, Houston, Texas 77030 USA; 6 Department of Molecular and Cell Biology, Baylor College of Medicine, Houston, Texas 77030 USA; 7 Department of Mechanical Engineering and Materials Science, Rice University, Houston, TX 77005 USA; 8 Department of Chemistry, Rice University, Houston, TX 77030 USA; 9 Department of Biomedical Engineering, University of Houston, Houston, 77204 TX USA

## Abstract

Surgical margin status in cancer surgery represents an important oncologic parameter affecting overall prognosis. The risk of disease recurrence is minimized and survival often prolonged if margin-negative resection can be accomplished during cancer surgery. Unfortunately, negative margins are not always surgically achievable due to tumor invasion into adjacent tissues or involvement of critical vasculature. Herein, we present a novel intra-operative device created to facilitate a uniform and mild heating profile to cause hyperthermic destruction of vessel-encasing tumors while safeguarding the encased vessel. We use pancreatic ductal adenocarcinoma as an *in vitro* and an *in vivo* cancer model for these studies as it is a representative model of a tumor that commonly involves major mesenteric vessels. *In vitro* data suggests that mild hyperthermia (41–46 °C for ten minutes) is an optimal thermal dose to induce high levels of cancer cell death, alter cancer cell’s proteomic profiles and eliminate cancer stem cells while preserving non-malignant cells. *In vivo* and *in silico* data supports the well-known phenomena of a vascular heat sink effect that causes high temperature differentials through tissues undergoing hyperthermia, however temperatures can be predicted and used as a tool for the surgeon to adjust thermal doses delivered for various tumor margins.

## Introduction

Surgical margin status in cancer surgery represents an important factor affecting the overall prognosis of the patient. The risk of adverse patient outcomes and surgical-margins recurrence is usually greatly minimized if the surgeon is able to achieve a grossly and pathologically negative margin during cancer surgery^1^. Unfortunately, there are many cancers for which negative margins cannot be surgically achieved at the time of diagnosis due to various factors, including tumor involvement of critical anatomical structures^2–12^. Such locally advanced invasion may constitute a contraindication to surgery, and if surgery is attempted, patients stand at high risk for early tumor recurrence and further disease progression.

Tumor involvement of major vasculature represents a perplexing problem that increases both surgical and oncologic risks for poor outcomes, with significant likelihood of a positive surgical margin^2–12^. This is seen in a wide range of cancers including, but not limited to, paragangliomas^5^, hepatocellular carcinoma^13^, pancreatic ductal adenocarcinoma (PDAC)^14, 15^, perihilar cholangiocarcinoma^2, 3^, neuroblastoma^6^, leiomyosarcoma^8^, retroperitoneal sarcoma^16^ and Kaposiform hemangioendothelioma^8^. Venous involvement can sometimes, but not always, be addressed by surgical resection and reconstruction of the vessels affected, such as in the case of hepatocellular carcinoma, which has invaded the portal vein, hepatic vein or inferior vena cava^7^. However, these procedures come with an increased risk to the patient^13^. PDAC^14, 15^, neuroblastoma^6^, Kaposiform hemangioendothelioma,^8^ gastrointestinal neuroendocrine tumors^17^, and metastatic squamous cell carcinoma^18^ represent some cancers that commonly display arterial involvement. Arterial resection and reconstruction represent an even greater risk and often represent a contraindication to surgery.

The work herein uses *in vitro* and *in vivo* models to investigate the use of applied hyperthermia to intra-operatively treat patients when a positive surgical margin is enountered. We use PDAC as a cancer model for these studies as PDAC commonly displays involvement with major mesenteric vessels, in particular the superior mesenteric artery (SMA)^14, 15^ (Figure S1A–C). Our method for applying hyperthermia was through a novel prototype device named the ‘CorleyWare’ device (CWD). The CWD is a resistive heating device designed to facilitate a uniform heating profile around the tumor and is based on the phenomenon of cancer cells being especially sensitive to hyperthermia^19^. Unlike conventional hyperthermia intraoperative techniques, such as RF ablation (standard RF ablation thermal dose is 70 °C for 5 minutes^20^) that are associated with coagulative necrosis and inflammation to healthy periablative tissues^20^, the CWD aims to expose cancer tissue to more mild hyperthermia over the tens of minutes timescale (41–46 °C for 10 minutes) to eliminate cancer progression after surgery whilst preserving healthy adjacent tissues. A schematic overview of the concept is highlighted in Figure S1D and the two versions of the device are depicted in Figure S2. Furthermore, we believe this form of intra-operative hyperthermia treatment may target a dangerous sub-population of cancer cells, namely cancer stem cells (CSCs)^21^, which are implicated in tumor resistance and recurrence. CSCs are defined as cells within a tumor that can self-renew and drive tumorigenesis. It is hypothesized that CSCs may generate tumors through stem cell processes of self-renewal and differentiation into multiple cell types. Although some studies have shown that certain agents, such as siRNA, can somewhat reduce CSCs populations^22, 23^, there are currently no approved treatments that specifically target CSCs, which contributes to slow advancements in patient outcome over the last four decades when an intravenous cytotoxic or biological agent approach has been taken.

In summary, we provide insight into the effects of mild hyperthermia on cancer, stromal and endothelial cells *in vitro* 2D monolayer settings, including CSCs renewability potential. We determine hyperthermia gradients in tumor tissue due to localized heating and subsequent intra-tumoral cellular damage due to hyperthermia *in vivo* murine and swine models. Finally, we validate our results with a simple mathematical model of hyperthermia dissipation in tumor encased SMA tissues when exposed to CWD heating.

## Results and Discussion

### The effect of hyperthermia on PDAC, Pancreatic stellate cells and Endothelial cells

**Figure 1:**
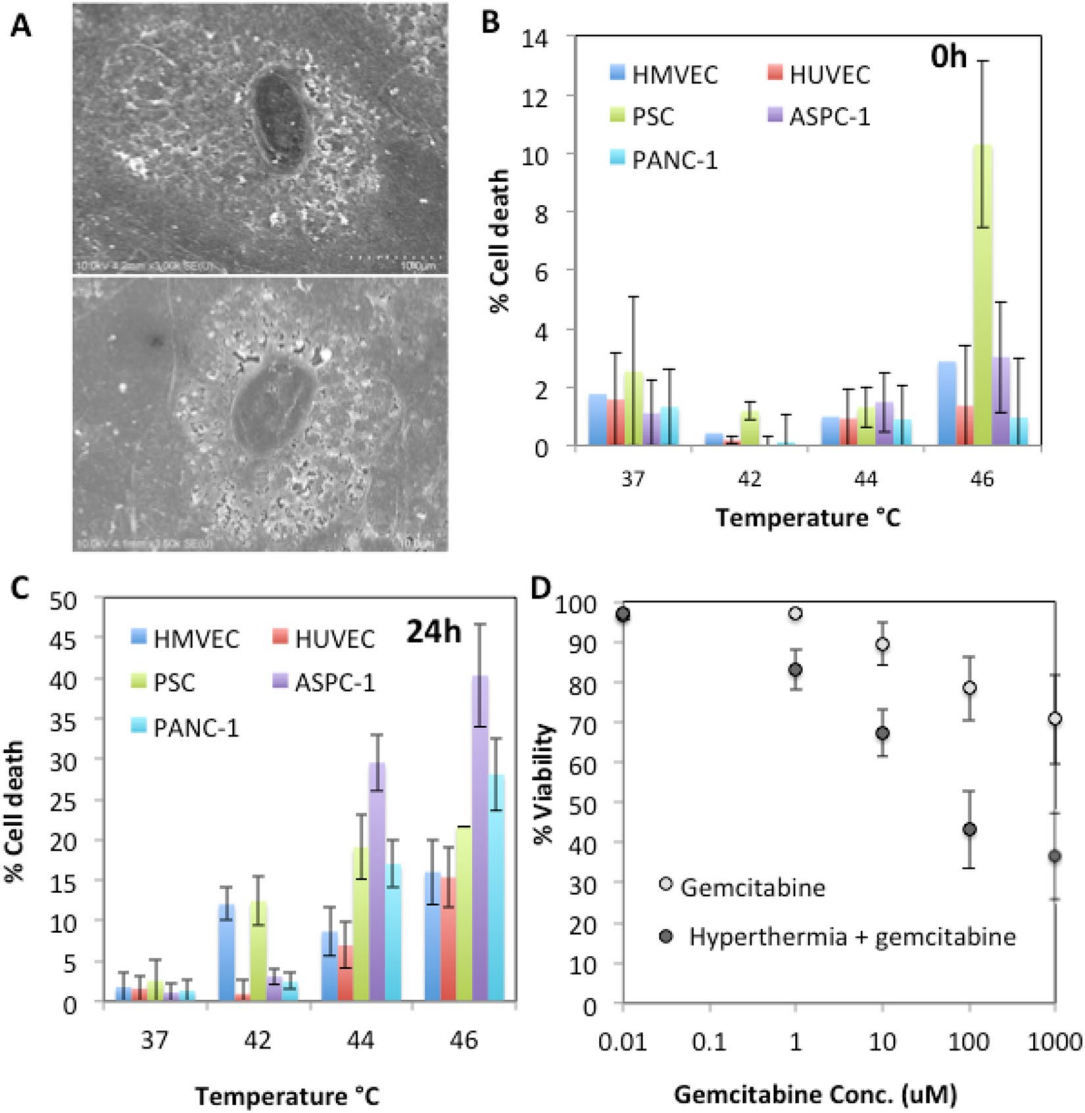
Heating and cell viability. (**A**) Morphological changes in HUVEC cells 24 h after hyperthermia (44 °C for 10 min) are not observed via SEM. (**B**) Percentage cell death of various cell lines of the pancreatic cancer microenvironment at 0 h after heat treatment at various temperatures (**C**) % cell death of various cell lines of the PDAC microenvironment 24 h after heat treatment at various temperatures (**D**) Viability of PANC-1 cells 48 h after gemcitabine exposure and hyperthermia pre-treatment (Thermal treatment consisted of 10 minute duration at desired temperatures (i.e. 37, 42, 44 and 46 °C, error bars represent standard deviation, experiment performed in triplicate).

*In-vitro* investigation was carried out to establish an approximate thermal dose that eliminated PDAC cell lines while minimizing the effects on healthy tissues. The CWD will be required to operate within an optimal thermal dose, where there is sufficient hyperthermia to enable tumor destruction while simultaneously limiting damage to healthy vascular tissue, as local thrombosis may occur leading to ischemia in the tissues downstream of the SMA and cause major complications in the patient. At the same time, weakening of the arterial wall can cause pseudoaneurysmal dilation and rupture, an equally catastrophic outcome. Initial *in-vitro* studies will allow the determination of thermal doses needed for the CWD application. The cell death induced by thermal doses of 42 °C, 44 °C and 46 °C for 10 minutes after an initial thermal increase from 35 °C which took 5 minutes were investigated (Fig. 1A).

The benefit of *in-vitro* population-wide high throughput cytotoxicity studies have previously been described^24^. This analysis was performed using numerous relevant cell lines to the PDAC-SMA environment and indicated that a thermal dose of 44–46 °C for 10 min would induce the greatest cytotoxicity in cancer cell lines while minimizing the effects seen in normal vascular endothelial cell lines (Fig. 1A–C). Activated pancreatic stellate cells (PSCs) were also included in the analysis as these cells are known to drive the dense stromal reaction often found in human pancreatic tumors^25^ and have been linked to tumor progression^26–30^. Other hyperthermia effects on PDAC cells, such as cytoplasmic retraction and alterations in cell metabolism have also previously been reported^31^. Importantly, we have found that hyperthermia pretreatment means an increase in susceptibility of cancer cells to chemotherapy (Fig. 1D). This is a clinically relevant finding as intraoperative hyperthermia administration via the CWD may induce the positive margin left after surgery to be more chemo-sensitive in later patient chemotherapy regimens. However, it is not currently known how long increases in susceptibility to chemotherapy due to hyperthermia pretreatment remain. Although these studies do not recapitulate the true biological environment, which includes arterial blood flow and the extra-cellular environment, they do provide some insight into the thermal dose required for greatest differential in cell death between malignant and healthy cells.

**Figure 2:**
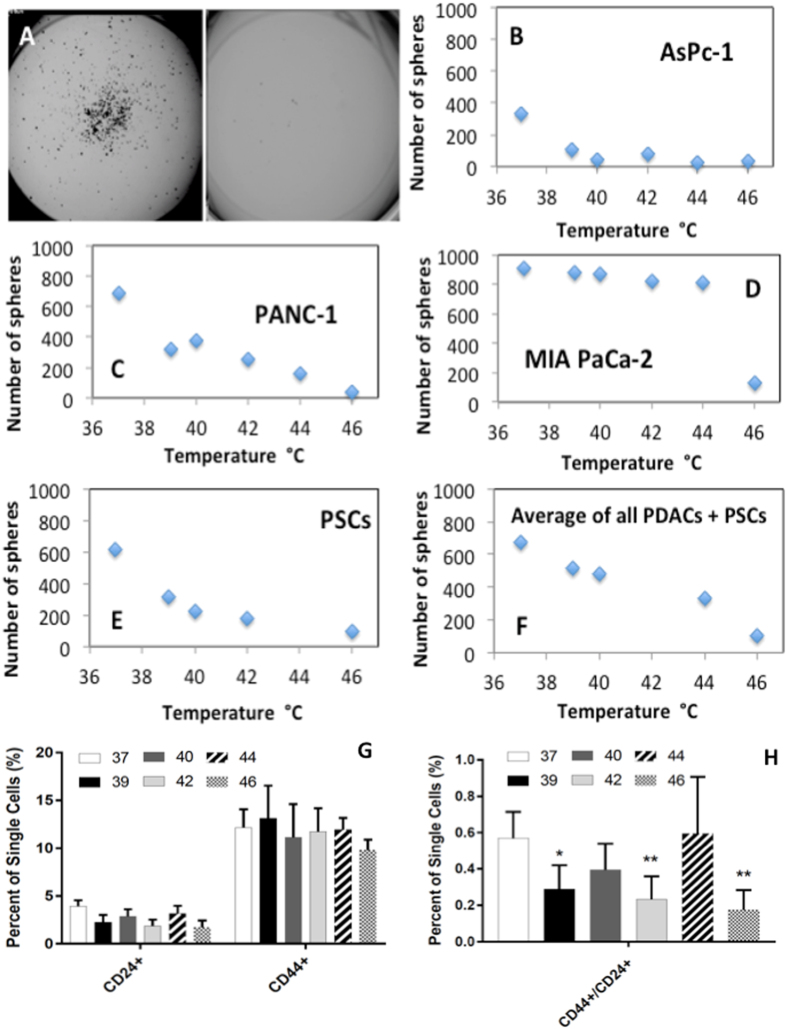
Hyperthermia decreases renewability of PDAC stem cells. (**A**) Brightfield image of PANC-1 cancer cells before treatment (left) and with heat treatment (46 °C for 10 minutes) (right) (**B**–**F**) the number of viable PDAC and PSCs spheres formed at 14 days after water bath heat treatment. G-H) CD24+ and CD44 CSC surface marker expression in PANC-1 cells after 37–46 °C hyperthermia exposure for 10 minutes.

In addition to the treatment of the bulk of the tumor remaining after surgery, we believe hyperthermia administered to PDAC cells encompassing the outer surface of the SMA will target specific cell populations that are particularly relevant to tumor drug resistance and regrowth, which include CSCs. Water bath heating of several cancer cell lines destroyed the viability and renewability potential of CSCs within these cancer cell populations (Fig. 2), as measured via tumor sphere forming assay and surface marker CD24 and CD44 analysis. The expression of CD24, CD44 surface markers have previously been associated with cancer cell stem-ness^32–34^, however the tumor sphere formation assay was chosen as the primary determination of CSC over surface marker expression in this study due to the potential of surface marker membrane binding sites being affected when exposed to hyperthermia and thus may lower their predictive power of cancer stem-ness. We have recently reported that hyperthermia in these ranges (37–46 °C) alter PDAC cells’ membrane and affects their ability to adhere to the cell culture substrate^35^. The tumor sphere formation assay is a functional test that determines the CSC behavior of the cell population despite what markers the cells may express on their membrane. This being said, we have included CD24 and CD44 surface membrane expression in one cancer cell line (PANC-1) and showed that CSC marker expression declined as thermal dose is increased (Fig. 2). Sphere formation 14-days after heat treatment was quantified to rule out the possibility of heat-treated CSCs remaining dormant for a length of time, only to develop at a later date, which prompts recurrence of a tumor. The tumor formation assay **(**Fig. 2 **)** indicates that a single heat treatment (41 °C for 10 mins) eliminates (>95%) of CSCs in PDAC cell populations, as it abolished the tumor sphere forming ability of the treated cells *in-vitro*. This effect was present in a wide range of different PDAC cell lines; AsPc-1, PANC-1, MIA-PaCa-2 as well as in non-malignant pancreatic stellate cells (PSCs). PSCs were included in this set of experiments as recent studies have alluded to PSCs playing an important role in promoting local growth of PDAC, facilitating regional and distant spread of PDAC cells^26, 27^, aiding PDAC immune-evasion^28, 29^ and facilitation of a CSC niche in PDAC, all of which have major impact on the progression of PDAC and play a role in its high recurrence rate. Alterations to PSCs may also play a role in halting PDAC tumor renewal after CWD treatment. Although responses observed were thermal dose dependent, effects were observed even at the lowest thermal doses.

All PDAC cell lines and PSCs showed a complete disruption of CSC viability when given a thermal dose of 46 °C for 10 minutes (Fig. 2). This indicates that collateral thermal damage to healthy tissues during heat treatment can be minimized as 46 °C for 10 minutes is not classed as ablation which causes inflammation and damage to healthy tissues surrounding the diseased loci.

We further investigated the effects of hyperthermia on PDAC cells by exploring the changes in protein expression induced by mild hyperthermia (41 °C for 10 mins) via the reverse-phase protein array (RPPA). The antibody panel used for the RPPA included 212 proteins covering major cellular signaling pathways involved in numerous aspects of cancer development and progression. The Student’s t-test was used to identify significantly differentiated levels of total or phosphoproteins with p-value < 0.05 and fold-change >1.25 (or <1/1.25). We discovered that mild hyperthermia induced the expression of 23 up-regulated proteins and 44 down-regulated proteins when compared to untreated cells (Figure S3A, Table S1). Further analysis using Ingenuity pathways analysis identified several significantly (Z-score >1 or <−1) associated canonical pathways, most of which were down-regulated after hyperthermia treatment (Figure S3B).

All hyperthermia-altered proteins are listed in Table S1, however full discussion on all altered proteins in the hyperthermia treated cells is outside the scope of this manuscript. Briefly, canonical pathway analysis, which correlates altered protein expression with specific pathways and disease states and bio-functionality highlighted many signaling pathways altered in hyperthermia treated cells (Figure S3B). Specifically, the most significant down-regulated pathways were pathways related to epidermal growth factor receptor (EGF), mitogen-activated protein kinase (MAPK), adenosine monophosphate-activated protein kinase (AMPK) and interleukin-8 (IL-8) signaling. The EGFR belongs to the ErbB group of receptor tyrosine kinases and previous studies have shown that it is involved in the pathogenesis and progression of various types of carcinoma^36^. The MAPK pathways are activated by various intra- and extra-cellular stimuli, such as cytokines, hormones, and cellular stressors such as endoplasmic reticulum stress and oxidative stress. MAPK pathways control a variety of cellular functions that include proliferation, differentiation, survival, and cell death. Deviation from the precise regulation of MAPK signaling pathways has been shown to play a role in the progression of various cancers^37^ and has been associated in immune evasion^38^ of melanoma cells. AMPK is a key energy sensor which is activated by increases in adenosine monophosphate (AMP)/adenosine triphosphate (ATP) ratio and/or adenosine diphosphate (ADP)/ATP ratio, and increases various metabolic pathways including mitochondrial biogenesis, fatty acid oxidation and glucose transport^39^. The activation of AMPK-autophagy pathway plays a role in drug resistance in colorectal cancer^39^. IL-8 is a chemo-attractant cytokine produced by various tissues^40^ and its up-regulation is implicated in pancreatic cancer invasion and metastasis^41^. p53 signaling was shown to be the most significantly up-regulated canonical pathway. The p53 homolog is crucial in multicellular organisms, where it prevents cancer formation, thus, functions as a tumor suppressor^42^.

To summarize, our *in vitro* data determines that a more mild hyperthermia, in the range of 46 °C in for 10 minutes is sufficient to cause cancer cell cytotoxicity, induce alterations in protein expression associated in various signaling pathways in PDAC cells and destroys CSC renewability potential.

### *In vivo* hyperthermia differential

Investigation into the heating kinetics of PDAC and vascular tissue provided valuable information to enable optimal thermal dose administration to the cancer layer whilst simultaneously minimizing unwanted damage to the intima and lumen of the SMA. CWD testing on *ex-vivo* and deceased *in-situ* porcine models determined the heat differential in ‘dry’ tissue, where blood flow is removed as a variable.

The porcine aorta was used in the *ex-vivo* experiments as it represents a good comparison to the human SMA due to a more consistent size. Without blood flow present, which is expected to increase the heat differential between outside surface and lumen of artery even further, there is a constant differential of approximately 2 °C (Figure S4) which is primarily due to the wall of the aorta.

In addition to a healthy aorta, we investigated the heat differential through an orthotopic murine PDAC tumor sample. The positive margin involved with the SMA will undoubtedly deviate from patient to patient, however we choose to investigate the heat differential across a 2 mm distance, as 2 mm represents a fairly realistic positive margin after surgery. An optical probe was place on the tumor boundary (Figure S4A) and another approximately 2 mm inside an *ex vivo* mouse orthotopic PDAC tumor (Figure S4B).

The quantification of heat differentials through artery and PDAC tumor tissue is essential for optimizing the thermal dose needed to destroy cancer cells throughout a 2–4 mm PDAC tumor positive margin while preserving the endothelium and smooth muscle found in the underlying artery. Failure to give an adequate thermal dose will mean low efficacy of the treatment whilst a thermal dose which is too high will cause damage to arterial tissues and could result in downstream ischemia in the gut or blood clotting, which are both dangerous to the patient and can be fatal. Figure S5A and B depict the experimental set-up and Figure S5D–F indicates the temperatures 2 mm inside an orthotopically grown PDAC tumor when the tumor-CWD boundary is heated to various temperatures. We find that a tumor-CWD boundary of 52 °C is necessary to achieve the proposed temperature gradient of at least 46 °C through a 2 mm thick tumor tissue.

**Figure 3:**
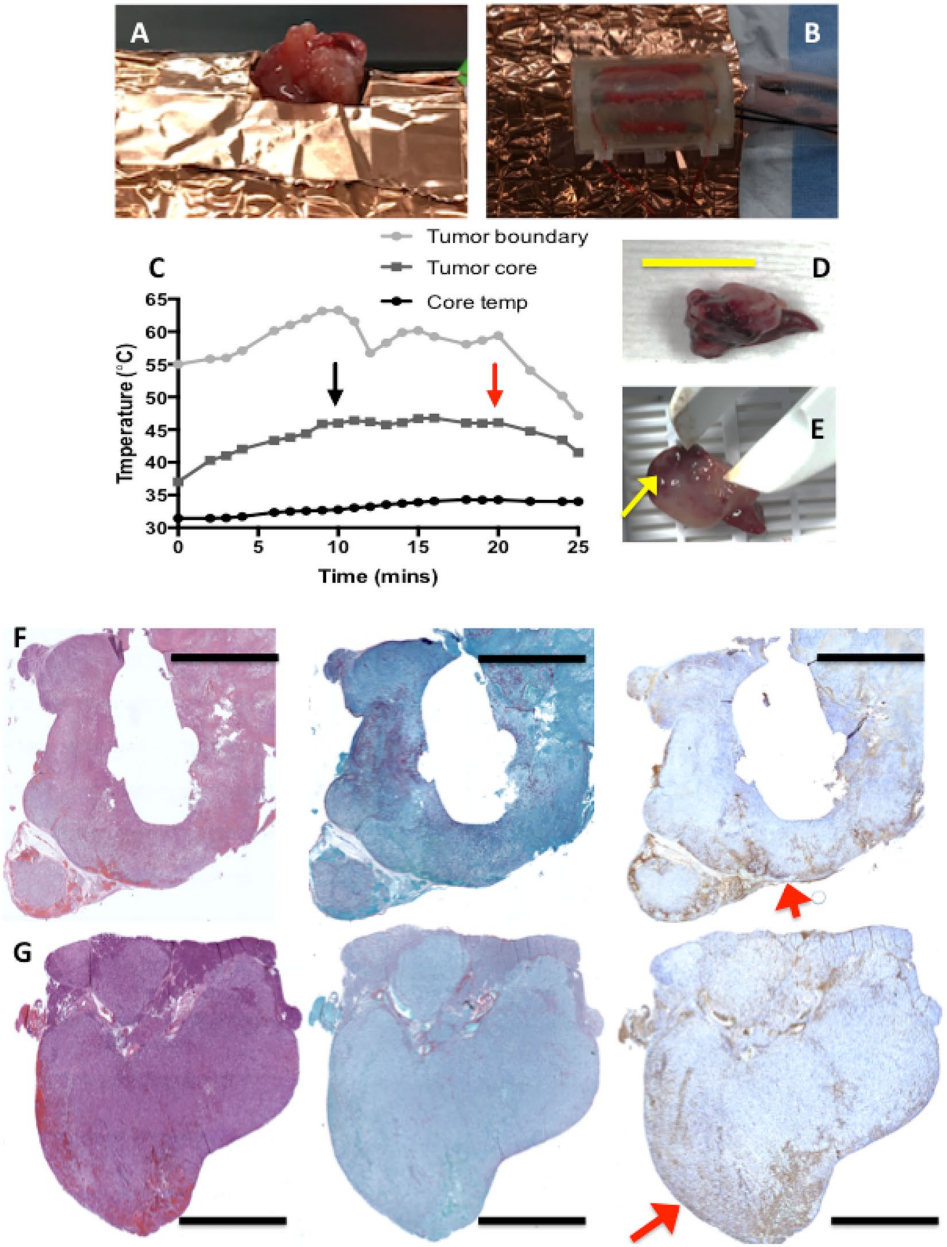
Tumor heat differential and cell death in *in vivo* murine PDAC model. (**A**) PDAC tumor is positioned for heat treatment (**B**) the heating device is placed onto the tumor (**C**) heat differential between tumor boundary and tumor core (mouse body core temperature is also displayed) (Black arrow depicts the point at which 46 °C in the tumor core was reached and the red arrow indicates the end of the treatment time when the device was removed﻿). (**D**) and (**E**) tumor after treatment (yellow scale bar = 2 cm, and yellow arrow represents heated side of tumor) (**F**) and (**G**) Row of histological cross-sectional micrographs of tumors treated with hyperthermia as described by Fig. 3A–E (from left to right, (H&E), picro Sirius and Cl-PARP histology stains, red arrows indicate heated surface, black scale bars = 2 mm).

We went on to investigate the heat differential in PDAC in a live mouse where blood flow was present within the PDAC tumor (Fig. 3). Once again, we placed an optical temperature probe on the surface of the tumor and inserted a second probe approximately 2 mm intra-tumorally. We also placed a rectal probe to record body core temperature to ensure the mouse was not subject to total body hyperthermia. As expected, we found the heat differential was increased (between 10–15 °C) when compared to the *ex vivo* tumor sample. The differentials seen here are substantial considering the tumor in this experiment is only fed by disorganized and small tortuous tumor vessels in a small animal model. Clinically, these differentials are underestimations when compared to the tumor under hyperthermia in PDAC human patients as the tumor tissue in the clinical setting will be proximal to a much higher blood flow from the SMA that is approximately 7 mm in diameter. Nevertheless, histological analysis of various tumor samples revealed excessive cell death between the border of the treated tumor and 2 mm inside the tumor as indicated by Cl-PARP histology staining (Fig. 3F,G). Collagenous extra cellular proteins, as indicated by picro sirius staining, did not show any significant alteration.

### Effect of the blood heat sink on heating dynamics

**Figure 4:**
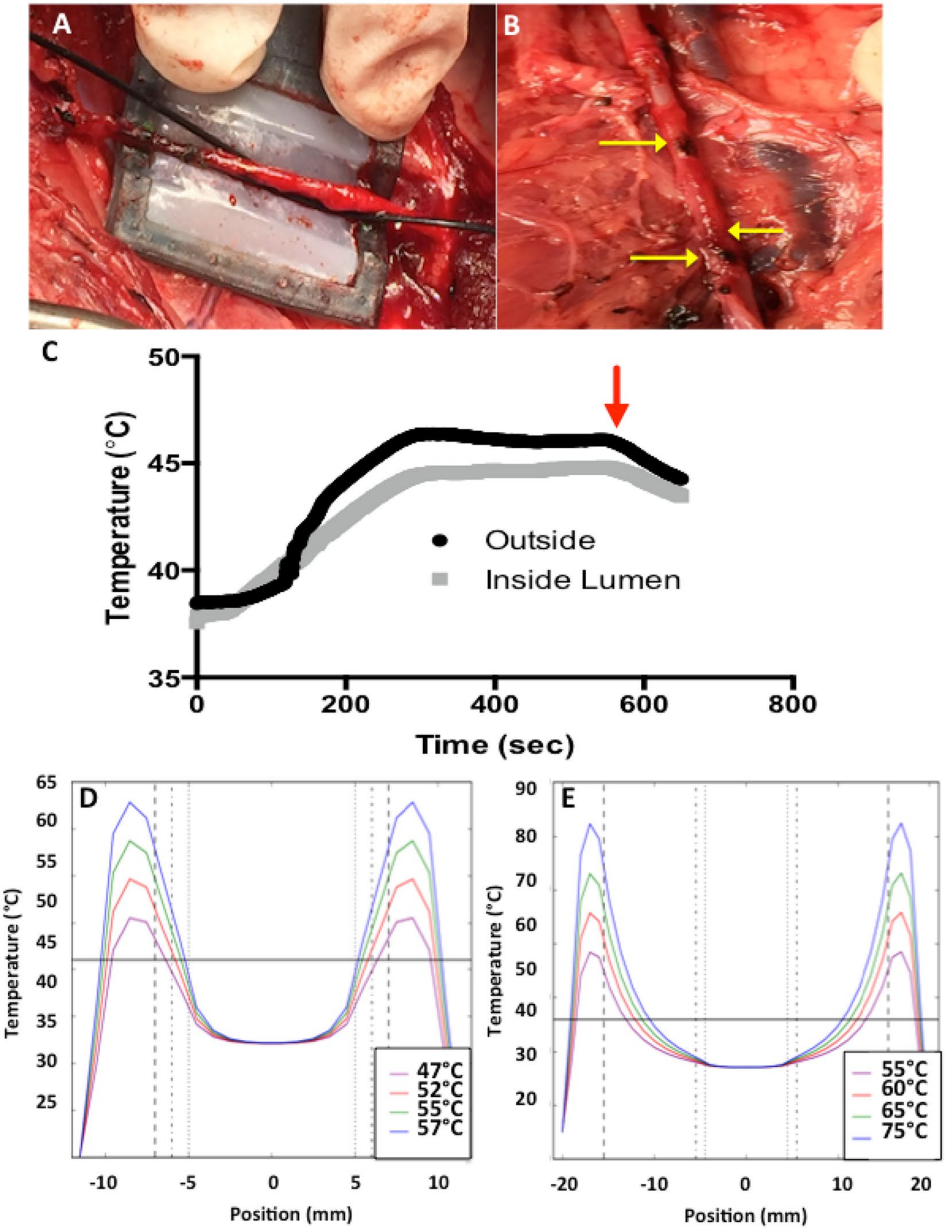
Effect of the blood heat sink on CWD heating dynamics in the femoral artery *in-vivo*. (**A**) CWD placement around femoral artery with insertion of a fiber optic temperature probe inside artery and another probe on the artery surface (probe inserted in direction of blood flow to minimize turbulence in blood flow inside artery) (**B**) femoral artery after hyperthermia treatment (yellow arrows indicate areas where cauterization was performed to skeletonize branches from main vessel) (**C**) Heating differential between the outside femoral artery surface (black curve) and inside the femoral artery lumen (grey curve). (Red arrow indicates time-point when hyperthermia was halted) (**D**) Simulation of tissue heating using the CWD on a 1 mm positive cancer margin and (**E**) simulation of tissue heating using the CWD on a 10 mm positive cancer margin. The solid horizontal line indicates 46 °C, the desired tissue temperature. The vertical dotted lines are the innermost edge of the blood vessel wall, the vertical dashed line is the outermost edge of the tissue and the vertical dash-dot line is the interface between the blood vessel and the tissue. Tumor tissue thicknesses are: (**A**) 1 mm (**B**) 10 mm.

Experiments regarding testing heating dynamics of the CWD-SMA system until now have been performed in *ex-vivo* SMA samples, in deceased *in-situ* porcine models or in *in vivo* murine models, which do not recapitulate the heavy pulsatile blood flow that would be present in human patients. A shortcoming of current various heating techniques in cancer therapy devices, such as radiofrequency ablation, is the limited performance adjacent to large blood vessels (diameter >3 mm). When the heating occurs near large vessels, the blood flow dissipates thermal energy away from the target tissue^43^. This is a heat sink effect that can change both the shape and maximum volume that can be treated. In fact, the distance of the blood vessels from the tumor determines the location of the maximal tissue temperature. As a result, tumors in the vicinities of large vessels are associated with high recurrence rates from incomplete thermal desctruction^44^. We believe we can mitigate these challenges by investigating the heating dynamics with specific consideration of blood flow characteristics along with the size of the patient and their SMA, and the thickness of the remaining positive margin enables a powerful tool to achieve insight into the heat dynamics within a given system. These details, in theory, will enable the surgeon to tune the CWD temperature to personalize the thermal dose given to a particular patient. However, we hypothesize that blood flow through the SMA lumen will be advantageous in this instance as it will increase heat differential between outside cancer layer and inside healthy vasculature. To explore this possibility we investigated the heating dynamics of the CWD when placed around the femoral artery (FA) in a live swine model to perform CWD testing when a high pulsatile blood flow is present and obtain temperature profiles of vessel interfaces as a function of time (Fig. 4). The FA was chosen for testing, as it is superficial for easier surgical access and also similar in size to the SMA in humans and is intended to provide proof of principle regarding the blood flow heat sink effect. An optic temperature probe was secured onto the outside surface of the artery, and a second probe was inserted into the lumen of the artery before the CWD was placed around the artery (Fig. 4A) and current was passed through the device to produce a localized thermal dose. This test confirmed the CWD could be accurately fitted to a vessel without major occlusion, which is particularly relevant because SMA occlusion can result in a downstream ischemic insult in the intestine^45^. The differential between the inside lumen and outside surface of the FA (artery wall is approximately 0.5–1 mm thick) when blood is flowing is ~1.5–2 °C when outside surface was heated to 46 °C (Fig. 4C). The vessel after heating showed minimal signs of thermal damage (Fig. 4B).

To complement our experimental findings, we developed a simple simulation to provide some information of the heating differentials seen through the whole tumor-vessel system (Fig. 4D,E). This simple simulation of the CWD utilized the Pennes bioheat transfer equation^46^ and used Sim4Life software (ZMT Zurich MedTech AG, Zurich, Switzerland)^47^. The model for all simulations consisted of a cylindrical blood vessel (inner diameter 10 mm, with 1 mm vessel walls), partially enclosed by a cylindrical section of pancreatic tumor tissue of thickness between 1–10 mm. Our aim was to heat the full thickness of the tumor tissue to at least 46 °C while limiting the temperature at the artery interface. The simulation displayed large thermal differentials even through small thicknesses of tissues. For instance, to achieve a temperature of at least 46 °C with a tumor tissue layer of only 1 mm, the CWD would have to heat the tumor boundary to at least 52 °C (Fig. 4D). For a 10 mm tumor layer, CWD tumor boundary temperatures in excess of 80 °C only achieved 46 °C through half of the tumor tissue (Fig. 4E).

**Figure 5:**
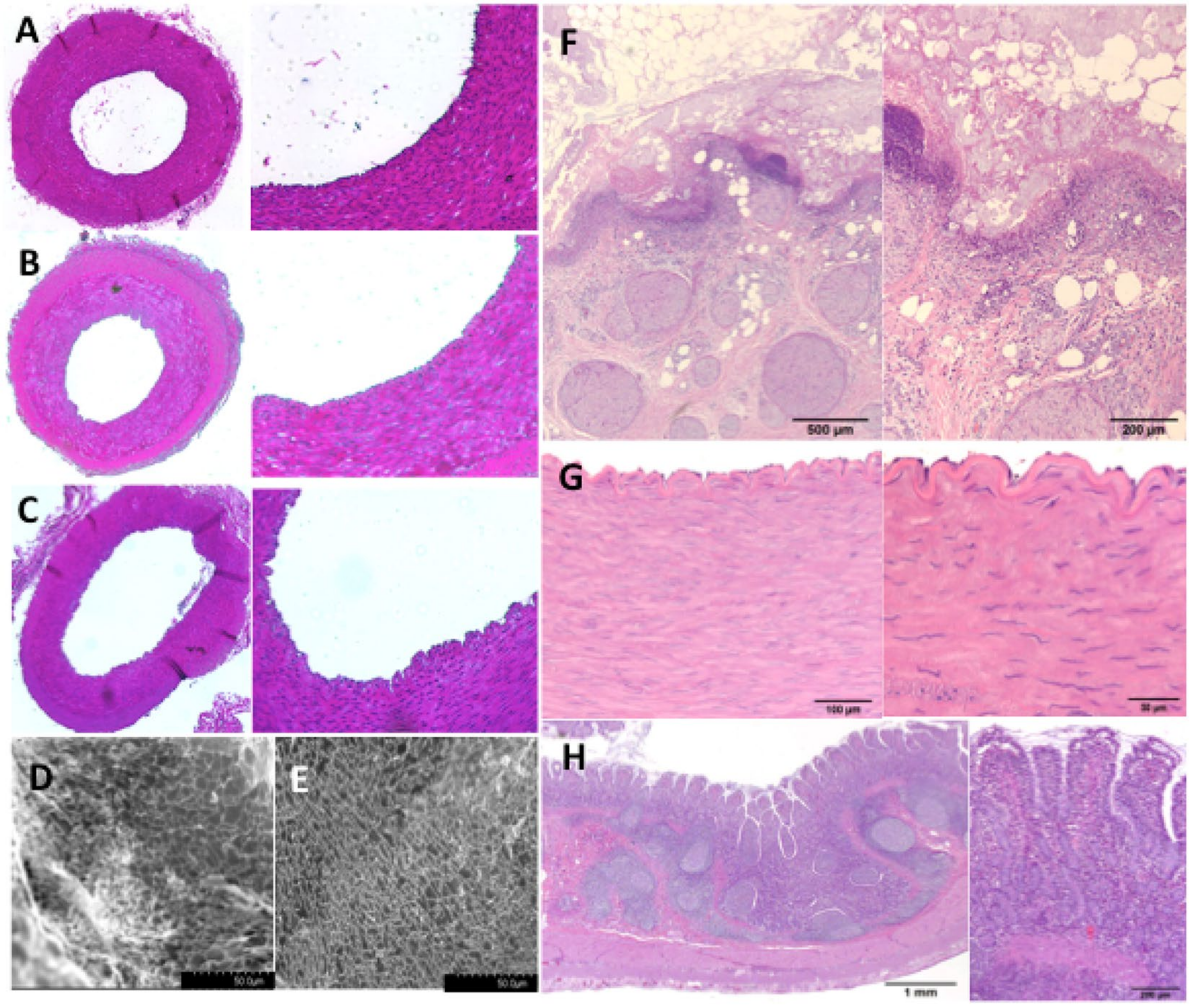
SMA Pathology after CWD treatment. Planar view of a femoral artery with corresponding zoomed in view of the endothelium (right). (**A**) Untreated femoral artery (**B**) femoral artery exposed to 46 °C for 10 mins and (**C**) femoral artery exposed to 55 °C for 10 mins. (**D**) Scanning electron micrograph of the tunica intima of an untreated and (**E**) 46 °C for 10 mins treated tunica intima of the femoral artery (**F**) Adipose tissue surrounding the treated portion of the artery (**G**) Muscle wall and endothelial layer of the treated portion of the artery (**H**) Small intestine (right hand images are zoomed in images).

The aim of the CWD is to deliver a thermal dose intra-operatively to the outside tumor layer that is sufficient enough to destroy cancer cells and to delay or eliminate recurrence of the tumor. It is essential that at the same time the prevention of significant damage to the underlying artery is achieved and hence the function of downstream tissues and organs can be maintained. Additionally, thrombosis or weakening of the heated artery needs to be avoided. The arterial viability following hyperthermia treatment was evaluated by histology following the excision of the FA straight after treatment. Figure 5A,B and C shows a planar view of an untreated, a CWD-treated FA and a FA that has undergone extensive heating, respectively, (55 °C for 10 mins) to act as a positive control. No obvious pathological features exist in the tunica intima, media or adventitia immediately after CWD treatment at this thermal dose. Alternatively, the positive control shows a ruffled endothelium and shrinkage in the width of the tunica media. SEM imaging performed on the tunica intima layer of the vessel showed no observable pathology (Fig. 5D,E).

The data thus far has not considered any delayed onset of pathological conditions relating SMA hyperthermia treatment, such as structural compromise of the vessel, ruffled endothelial layers or downstream abdominal ischemia. For this consideration, we exposed the anterior mesenteric artery (AMA, which is the quadruped analogue of SMA) of a live pig to 41 °C for 10 minutes and a necropsy was scheduled for 48 h after treatment. An inspection of the AMA, surrounding tissues, including the proximal vessels and the small intestines found no obvious damage to any of the organs immediately after hyperthermia treatment. The animal was monitored throughout the day and into the evening by veterinary staff. A heat lamp was provided to aid in thermoregulation after surgery. Postoperative antibiotics were administered (5 mg/kg enrofloxacin IV twice, 5 mg/kg ceftiofur IM once, 1 gram cefazolin IV once, 1 L of lactated ringers saline IV twice, 60 mL D5W IV once) in the time between surgery and necropsy. At the 48 h time-point, the animal was euthanized after sedation. The animal was euthanized after 48 h because it showed signs of distress and abdominal discomfort, which on gross pathologic examination was due to an inadvertently retained laparotomy sponge at the initial surgical procedure. There was post-operative peritonitis from the foreign body but there was no observed evidence of ischemic necrosis in the stomach, small intestine, or large intestine. The AMA appeared patent with no observed thrombosis or dilation; all bowel appeared viable without evidence of vascular compromise (Figure S6, Fig. 5). There was an extensive foreign body reaction and localized fibrinopurulent peritonitis with an accumulation of a large amount of ascitic fluid in the peritoneal cavity as a result of the extensive manipulation of the abdominal organs and the dissection and isolation of the AMA, as well as fibrinous adhesions between loops of intestine commensurate with the recent laparotomy and reaction to the foreign body.

Histological analysis revealed acute marked fibrinopurulent inflammation and coagulative necrosis around the isolated treated segment of the artery that is consistent with a surgical procedure that reportedly involved extensive manipulation of the abdominal viscera (Fig. 5). The isolated segment of the artery consisted of a centrally located artery surrounded by a 4–6 mm thick zone of soft tissue composed of numerous peripheral nerves, mesenteric adipose tissue, and fibrovascular tissue. The coagulonecrotic tissue observed microscopically was located primarily in a layer on the surface of the treated portion of the artery and is likely a result of thermal necrosis associated with the thermal therapy applied to the artery (Fig. 5F). The coagulative necrosis appears to primarily affect the mesenteric adipose tissue resulting in saponification of the adipose tissue. There was coagulative necrosis of a few isolated peripheral nerve bundles near the treated surface of the tissue surrounding the mesenteric artery. Importantly, there was no observed damage to the AMA in the center of the bundle of treated tissue and neither in the intestines (Fig. 5G and H).

## Discussion

Vascular and in particular arterial invasion, is a key aspect in the resectability of a range of tumors, and therefore often dictates a patient’s prognosis and survival. Here, we describe the functionality of an innovative new device we have developed, named the CorleyWare device, to treat tumors with arterial involvement. The long-term goal of the device is to provide an intra-operative tool to deliver localized, mild and controlled hyperthermia to cancer margins that cannot be surgically resected due to them laying close to an unresectable vessel. We used PDAC as a model for our studies as it commonly associates with mesenteric vasculature during its local advancement. *In vitro* data suggests that mild hyperthermia, in the range of 46 °C for 10 minutes is an optimal thermal dose to induce high levels of cancer cell death when compared to non-malignant cells and eliminate the renewability function of CSCs. *In vivo* and *in silico* data supports the well-known phenomena of a blood heat sink that causes high temperature differentials through tissues undergoing hyperthermia. Additionally, the tissues themselves, especially tumor tissue, have high temperature differentials over relatively short distances representing common positive margins that may be due to the tissue characteristics such as the dense stromal reaction found in PDAC.

We believe hyperthermia treatment of the positive margin left on vessels such as the SMA during surgery in patients whose tumors are to date considered borderline or unresectable may allow a longer period of time between surgery and tumor recurrence, hence increasing patient recovery time before being able to commence chemotherapy regimens and improving the probability of achieving surgical result commensurate with curative intent. However, we acknowledge that this will need to be proven clinically in future trials.

Additionally, we propose that the heat differential provided by blood flow in the vessel will enable a protective effect for the inside lumen whilst the outside cancer layer is heated. This will mean that the outside cancer layer can possibly be exposed to higher thermal doses increasing kill efficacy during hyperthermia treatment. The effect of the blood heat sink may be further enhanced with the use of a localized blood cooling aid upstream of the device and/or a splanchnic vasodilator to increase arterial blood flow; however these were not tested herein. Previous device designs encased larger portions of the artery and we noticed that the slimmer design enabled the blood heat sink to be maintained for longer periods (Data not shown). We speculate this is due to the slim design of the later device meant that blood had a shorter distance to travel under hyperthermia and hence would not have a time to substantially increase in temperature and would therefore conserve the heat sink effect to a greater degree.

Our simulations of a simplified model of pancreatic tumor tissue surrounding a blood vessel predicted a large thermal differential through a relatively thin section of tissue. These large thermal differentials were validated by our *in vivo* and *ex vivo* experiments. Future simulations will be more sophisticated, including pulsed arterial flow, variable heart rate, and explicit simulation of blood flow. These simulations will provide an important tool for the surgeon when deciding the thermal doses warranted for patients of different sizes and weights with varying positive margin thicknesses.

Lines of future exploration should involve techniques to increase the blood cooling effect by introducing a localized cooling aid such as a cooling pack (33 °C and 36 °C, as anything below 30 °C may introduce vasoconstriction) upstream of the tissue undergoing hyperthermia, cooling the encased artery as the device is heating the outside cancer layer and thus hypothetically increase the heat differential between the cancer layer outer surface and the lumen of the vessel. The efficacy of the device may be improved by the incorporation of chemotherapeutic drugs into the inner surface of the device. Drugs can be delivered homogeneously on the tumor section encompassing the artery via the use of micro-needles or sponge delivery system, such as intercalated chemotherapeutics into various polymers such as PLA or biocompatible silk scaffolds. Finally, it is noteworthy that while the work herein used PDAC as a model cancer, we foresee this device being used to treat other cancers where resection is not accessible.

## Methods

### Ethic statement

All experiments were performed after approval of the Institutional Animal Care and Use Committee (IACUC) of Baylor Collage of Medicine (No. AN-6448 and AN-7062) and followed established protocols.

### Cell lines

Human PDAC lines, PANC-1 and AsPc-1, cells were obtained from American Type Culture Collection (ATCC). PANC-1 and AsPc-1 cells were maintained in DMEM with 10% FBS. Human umbilical vein endothelial cells (HUVEC) (Lonza, Walkersville, MD, USA) were maintained in vascular cell basal medium supplemented with 0.2% bovine brain extract, 5 ng/mL rh EGF, 10 mM L-glutamine, 0.75 units/mL heparin sulfate, 1 µg/mL hydrocortisone hemisuccinate and 2% fetal bovine serum and 50 µg/mL ascorbic acid.

Human microvascular endothelial cells (HMVEC) (Lonza, Walkersville, MD, USA) were incubated in endothelial cell basal medium (Lonza) supplemented with endothelial cell growth medium (EGM) microvascular bullet kit containing 25 mL FCS, 0.5 mL hEGF, 0.5 mL hydrocortisone, 0.5 mL gentamicin, 0.5 mL bovine brain extract (Lonza). Cells were grown to confluence and when needed harvested with trypsin-EDTA (Lonza). All cells lines were maintained in a 95% humidified atmosphere of 5% CO_2_ at 37 °C and 2% penicillin-streptomycin solution was added to the media of all cell lines.

Human pancreatic stellate cells (PSCs) were provided by Dr. R. Hwang (UT-MD Anderson Cancer Center, Houston TX). Fresh tissue was obtained from residual PDAC specimens from patients undergoing primary surgical resection at The University of Texas M. D. Anderson Cancer Center. Informed consent was granted for the harvesting of human tumor tissue samples to isolate pancreatic stellate cells. All human samples were obtained in accordance with the policies and practices of the Institutional Review Board of The University of Texas M. D. Anderson Cancer Center. The PSCs were isolated and prepared as previously described^30^ Briefly, PSCs were prepared by the outgrowth method^48^. Fresh tissue was obtained from residual PDAC specimens from patients undergoing primary surgical resection at The University of Texas M. D. Anderson Cancer Center. All human samples were obtained in accordance with the policies and practices of the Institutional Review Board of The University of Texas M. D. Anderson Cancer Center. Tumor samples were minced and seeded in six-well plates containing 15% FCS/DMEM, l-glutamine (2 mmol/L), penicillin/streptomycin, and amphotericin. Five days later, cells were able to grow out from the tissue clumps. When PSCs grew to confluence, cells were trypsinized and passaged at a 1:3 ratio. Cell purity was determined by immunohistochemistry for alpha-SMA, vimentin, and desmin, as well as morphology (spindle-shaped cells with cytoplasmic extensions) and positive staining with Oil Red O (lipid inclusions were visualized). Further, PSC derived from the pancreatic tumor of a patient who had received no prior therapy before surgery were immortalized using lentiviral vector with human telomerase (hTERT) or SV40 large T antigen (TAg) through plasmids containing TAg (pHIV7-CNPO-TAg) and hTERT (pHIV7-CNPO-hTERT) as previously described in details^49^. PSCs carrying the hTERT or SV40-T were selected in 1 to 3 mg/mL G418 for 3 weeks (Invitrogen). Cells were maintained in DMEM with 10% FBS at 37 °C in a humidified atmosphere of 5% CO_2_.

### *In-vitro* time-resolved cytotoxicity testing

Cytotoxicity was quantified using DNA-binding DRAQ7 (Biostatus Ltd, UK) staining, which is a far-red fluorescent dye. Cells were seeded at a concentration of 100,000 cells per well in Greiner Bio One Cellstar® tissue culture 6-well plate for 24 h. Plates were then placed into a water bath and heated to the given thermal dose. An optical probe was inserted into a cell free well that contained 2 ml of media so that accurate thermometry of the plate bottom could be achieved. After 10 mins of water bath hyperthermia, 3 µM concentration of DRAQ7 was placed in the cell medium before being gently pipetted to ensure thorough mixing. High throughput microscopy was performed within 10 min of adding the DRAQ7 using the ImageXpress Micro XL microscope (GE Healthcare, USA). Images were obtained which included 10,000 cells at 0 h and 24 h time points in triplicate for statistically robust findings. Cells were imaged at 20x magnification in two wavelengths: the DRAQ7 (Cy5, Absorbance 600 nm emission 697 nm) and the bright-field wavelengths. At the end of the time-lapse experiment the cells were placed in 52 °C water for 10 minutes to induce 100% cell death before an additional set of images was collected, from which a measure of the total number of cells in each well was obtained. Cell death was quantified by the automated measure of the DRAQ7 signals at 0 and 24 h time points using MetaXpress 5.3.0.5 and MetaXpress PowerCore 1.3.0.3 software (Molecular Devices, USA).

### Reverse-Phase Protein Array Assay

Reverse phase protein array (RPPA) assays were performed as described previously^50, 51^ with minor modifications. Protein lysates were prepared from the cell culture samples with Tissue Protein Extraction Reagent (TPER; Pierce) supplemented with 450 mM NaCl and a cocktail of protease and phosphatase inhibitors (Roche Life Science). Protein lysates at 0.5 mg/ml of total protein were denatured in SDS sample buffer (Life Technologies) containing 2.5% 2-mercaptoethanol at 100 °C for 8 min. Protein lysates were arrayed onto nitrocellulose-coated slides (Grace Bio-labs, Bend, OR, USA) using an Aushon 2470 Arrayer (Aushon BioSystems, Billerica, MA, USA) with an array format of 960 (experimental and controls) lysates per slide with each sample spotted as technical triplicates (2,880 spots per slide). Slides were blocked for 1 h with I-Block reagent (Applied Biosystems) followed by a 15 min incubation with Re-Blot reagent (Dako) and were loaded on an automated slide stainer Autolink 48 (Dako, Carpinteria, CA, USA) for incubation with primary antibodies. Antibody binding was detected by fluorescence with a Vectastain-ABC Streptavidin–Biotin Complex (Vector, PK-6100) followed by incubation with the TSA-plus Biotin Amp Reagent diluted at 1:250 (Perkin Elmer, NEL749B001KT) and a 1:50 dilution of LI-COR IRDye 680 Streptavidin (Odyssey) as the detection probe. The total protein content of each spotted lysate was assessed by fluorescent staining with Sypro Ruby Protein Blot Stain for selected subsets of slides (Molecular Probes). Fluorescent-labelled slides were scanned on a GenePix AL4400 scanner, and the images were analyzed with GenePix Pro 7.0 (Molecular Devices). For normalization, raw image intensity of each spot was subtracted from that of negative controls and then divided by total protein values. Tumors with different genetic backgrounds were analyzed separately. Of the 212 validated antibodies included in the RPPA, 143 antibodies detect total protein and 69 detect specific phosphorylated states known to be markers of protein activation. The validated antibodies represent proteins in various signaling pathways and cell functional groups including growth factor receptors, cell cycle, cell proliferation, apoptosis, EMT, stem cells, DNA damage, cell stress, autophagy, cytokines, protein translation and gene transcriptional activators and repressors. For a complete list of validated antibodies see https://www.bcm.edu/centers/cancer-center/research/shared-resources/antibody-based-proteomics. Significantly differentiated antibodies between experimental groups were determined using a cutoff of P < 0.05 (by two-sided Student’s t-tests) and FC (fold change) > 1.25 (or < 1/1.25).

### Pancreatic cancer stem cell viability/renewability assays

Cells were seeded at a concentration of 100,000 cells per well in Greiner Bio One Cellstar® tissue culture 6-well plate for 24 h. Plates were then placed into a water bath and heated to the target thermal dose. An optical probe was inserted into a cell free well that contained 2 ml of media so that accurate thermometry of the plate bottom could be achieved. After 10 mins of water bath hyperthermia, the media was collected and the cells were trypsanised before being added to the supernatant media and spun down. For the tumor sphere-forming assay the cells were reseeded in six-well ultra-low attachment plates (Corning) with 2 mL of serum-free mammosphere medium. Fresh mammosphere medium was added to the wells every 3 days. The number of viable PDAC spheres at 14 days were imaged using ImageXpress Micro XL microscope (GE Healthcare, USA) and counted using MetaXpress 5.3.0.5 software (Molecular Devices, USA).

For the surface marker expression analysis, APC-labeled mouse anti-human CD44 (clone G44–26) and FITC-labeled mouse anti-human CD24 (clone mL5) were both purchased from BD Pharmingen (San Diego, CA). 7-AAD Viability Staining Solution was purchased from eBioscience (San Diego, CA). PANC-1 cells were removed from flasks using Enzyme Free Cell Dissociation Buffer (ThermoFisher) and rinsed in FACs buffer (PBS + 2% FBS) prior to staining. Cells were then stained for surface antigens according to the antibody manufacturer’s instructions. Following the last rinse to remove any free antibody, cells were suspended in 200 µL PBS and 5 µL 7-AAD viability stain was added 5–15 minutes prior to analysis on a Attune NXT flow cytometer (ThermoFisher). Analysis was performed using FlowJo version 10. Single color and unstained controls were used for proper gating and compensation.

### Scanning electron microscopy (SEM)

Cells were fixed by washing thrice with 0.1 M sodium cacodylate buffer (CDB) followed by incubation in 2.5% glutaraldehyde for 25 min at room temperature. Next, cells were washed twice in 0.1 M CDB and subjected to an ethanol series for dehydration. The cells were then incubated in 1:1 t-butanol:ethanol mixture for 5 min and mounted on carbon tape upon an SEM stub. Immediately before imaging the samples were sputter coated with 50% platinum 50% palladium at a thickness of 5.0 ± 0.2 nm to ensure good electrical conductivity.

### Generation of orthotopic PDAC tumors in mice and hyperthermia administration

Animal studies were performed in accordance with the guidelines of the Animal Welfare Act and the Guide for the Care and Use of Laboratory Animals based on approved protocols by Baylor College of Medicine Institutional Animal Care and Use Committee (IACUC). Mice were kept in standard housing conditions and diet with free access to water. Orthotopic pancreatic tumors were grown in 6–7 week old athymic nude (FOXn1 nu) female mice (Harlan Sprague Dawley, USA). Under sterile conditions a 1cm  incision was made in the left flank to expose the pancreas. PANC-1 cells (1 × 10^6^ in 40 µL of 1:1 PBS:Matrigel) were injected into the pancreas. The abdominal wound was closed in two layers. Tumors were allowed to grow for 6 weeks before being used for experimentation.

The CorleyWare device depicted in Figure S2A was used for *ex vivo* measurement of heating differentials and tumor damage due to hyperthermia. Tumor were excised from the mice after euthanization and then exposed to various thermal doses with optical temperature probes place on their surface and within their core before immediate fixation in 4% formaldehyde for histology preparation. The experimental set up of the tumor, CWD and temperature probes is shown in Figure S5A and B.

The measurement of *in vivo* heat differential and tumor damage due to hypethermia involved non-survival surgery where under anesthesia the tumor was gently removed from the abdomen of the mouse before probes were positioned on the tumor surface and into the core of the tumor. Hyperthermia involving various target thermal doses was then administered. The CorleyWare device depicted in Figure S2A was used for *ex vivo* measurement of heating differentials and tumor damage due to hyperthermia and the experimental set up involving the position of the tumor, mouse, temperature probes and CWD is shown in Fig. 3A and B. After the treatment, the peritoneum and skin layers were gently closed and the mouse was kept sedated for two hours under anesthesia before being euthanized. The tumor was then excised and immediately fixed in 4% formaldehyde for histology preparation.

### Large Animal Models

Female Yucatan miniature swine (Sinclair Bioresources; Auxvasse, MO) were housed and raised according to Institutional Animal Care and Use Committee (IACUC) guidelines at the Baylor College of Medicine. No special diet was given. At the time of Corleyware testing, pigs were 12 months of age and weighed 70–75 kg.

For femoral artery heat differential measurements, a Yucatan miniature swine was sedated and anesthetized without complications for non-survival surgery. Their femoral artery was exposed for the delivery of a localized thermal treatment. Hyperthermia was administered using the CorleyWare device depicted in Figure S2A and the experimental set up involving the position of the artery, temperature probes and CWD is shown in Fig. 4A. All animals were stable throughout the procedure.

For survival surgery when investigating any delayed onset of pathology associated with hyperthermia administration to the SMA, a female Yucatan swine was sedated and anesthetized without complications. The SMA was exposed and further skeletonized before being given a localized thermal dose of 41 °C for 10 mins using the CWD depicted in Figure S2C. This version of the device is used in anatomical locations were space is minimal. The experimental set up involving the position of the SMA and the CWD is shown in Fig. 3A and B. Temperature probes were not needed during this procedure due to this version of the device possessing an in-built temperature sensing surface thermocouple. Pain medications (75 mcg/hr fentanyl patch and 0.4 mg/kg Meloxicam SQ) and peri-operative antibiotics were provided (1 gram Cefazolin IV). The animal stable throughout the procedure with 1300 mL of IV LRS administered. A local anesthetic (5 mL lidocaine, 5 mL mL bupivacaine and 5 mL saline) was injected after closure. The swine was euthanized 48 h after the procedure, where a gross pathological assessment and a subsequent histological assessment of relevant tissues were performed.

### Immunohistochemical evaluation

Tissue samples were fixed in formalin and processed in paraffin blocks and sectioned using standard techniques. Tissue slides were stained with H&E, Verhoeff-Van Gieson (Elastic fibers) and Cleaved PARP (apoptosis). Histology slides were imaged using Nikon Eclipse TE2000-U microscope fitted with a Nikon digital sight DS-Fi1 video camera. All slides were imaged at a fixed 167 ms exposure time.

### Computer simulation of CWD

Simple simulations of the Corleyware device were performed using the software Sim4Life (ZMT Zurich MedTech AG, Zurich, Switzerland)^47^. The model for all simulations consisted of a cylindrical blood vessel (inner diameter 4.5–7 mm, with 1 mm vessel walls), partially enclosed by a cylindrical section of pancreatic tissue of thickness 1–10 mm. The Pennes Bioheat Transfer Equation^46^ was used for all simulations:

1 $$\rho s\frac{\partial T}{\partial t}=\nabla \cdot k\nabla T-{\rho }_{bl}{s}_{bl}{w}_{bl}(T-{T}_{bl})+{Q}_{m}$$

where *T* is the temperature of the tissue of interest, *t* is time, *ρ* is the density, *s* is the specific heat, *k* is the thermal conductivity, and *Q*_*m*_ is the rate of metabolic heat production for a specific tissue type. The constants *ρ*_*bl*_, *s*_*bl*_, and *w*_*bl*_ are the density, specific heat and perfusion rate of blood through the tissue, and *T*_*bl*_ is the temperature of blood. Note that the electric field heating term of the Pennes equation was omitted since it was not needed in this case. We also did not consider vasodilation of the artery in the simulation as during pig experiments we were treating a small portion of the artery (treated areas were ~3 cm in length) and no recordable vasodilative reaction of the artery were observed during surgery along with no decrease in heart rate due to decreased vascular resistance. Additionally, in mice experiments, core temperatures remained constant throughout the experiments and PDAC tumors were hypovascularized. Values for all physical constants were taken from the IT’IS Database for Thermal and Electromagnetic Parameters of Biological Tissues^52^. For all simulations, blood temperature was fixed at 37 °C and the background temperature was set to 25 °C. Flow of blood through the SMA was not explicitly modeled. Instead, blood flow was treated as constant, not pulsed, flow by the blood flow term of the Pennes equation:

2 $$({\rho }_{bl}{s}_{bl}{w}_{bl}(T-{T}_{bl})).$$

The Pennes equation was also used for non-biological materials. If the blood perfusion and metabolic terms are set to zero, the Pennes equation is reduced to the three-dimensional heat equation^53^. Heating of the Corleyware device was achieved by using a non-zero rate of metabolic heat production (*Q*_*m*_), where the value of *Q*_*m*_ was chosen to produce the desired temperature.

## Electronic supplementary material

Supplementary information
