# Supplementary material for: A new mild hyperthermia device to treat vascular involvement in cancer surgery
Source: Sci Rep. 2017 Sep 12;7:11299. doi: 10.1038/s41598-017-10508-6 (PMC5595878; doi:10.1038/s41598-017-10508-6)
Supplement: Supplementary file 1 — Supplementary information [file 41598_2017_10508_MOESM1_ESM.doc]

**A new mild hyperthermia device to treat vascular involvement in cancer surgery**

## *Matthew J. Ware^1^, Lam P Nguyen^1^, Justin J. Law^1^, Martyna Krzykawska-Serda^1,2^, Kimberly M. Taylor^1^, Hop S. Tran Cao^1^, Andrew O. Anderson^1^, Merlyn Pulikkathara^1^, Jared M. Newton^3,4^, Jason C Ho^1^, Rosa Hwang^5^, Kimal Rajapakshe^6^, Cristian Coarfa^6^, Shixia Huang^6^, Dean Edwards^6^, Steven A. Curley^1,7^# and Stuart J. Corr,^1,8,9^#*

^1^Department of Surgery, Baylor College of Medicine, Houston, TX, 77030, USA

^2^Faculty of Biochemistry, Biophysics and Biotechnology, Jagiellonian University

Gronostajowa 7 St., Kraków, 30-387, Poland

^3^Department of Otolaryngology-Head and Neck Surgery, Baylor College of Medicine, Houston, TX, 77030, USA

^4^Interdepartmental program in Translational Biology and Molecular Medicine, Baylor College of Medicine, Houston, TX, 77030, USA

^5^ Department of Surgical oncology, MD Anderson, Houston, Texas, 77030, USA

^6^Department of Molecular and Cell Biology, Baylor College of Medicine, Houston, Texas, 77030, USA.

^7^Department of Mechanical Engineering and Materials Science, Rice University, Houston, TX, 77005, USA

^8^Department of Chemistry, Rice University, Houston, TX, 77030, USA.

^9^Department of Biomedical Engineering, University of Houston, Houston 77204, TX, USA.

# Joint senior author

**CORRESPONDING AUTHORS:**

**Dr. Stuart Corr,**

**Department of Surgery,**

**Baylor College of Medicine**

**One Baylor Plaza,**

**ABBR R515,**

**Houston**

**Texas, 77030**

**Correspondence to Stuart.Corr@bcm.edu**

**Dr. Steven Curley,**

**Surgery/Surgical Oncology Division**

**Baylor College of Medicine**

**One Baylor Plaza,**

**Houston**

**Texas, 77030**

**Correspondence to Steven.Curley@bcm.edu**

**KEYWORDS:** Pancreatic Cancer, superior mesenteric artery encasement, hyperthermia, surgery

## 7.0 Supplementary


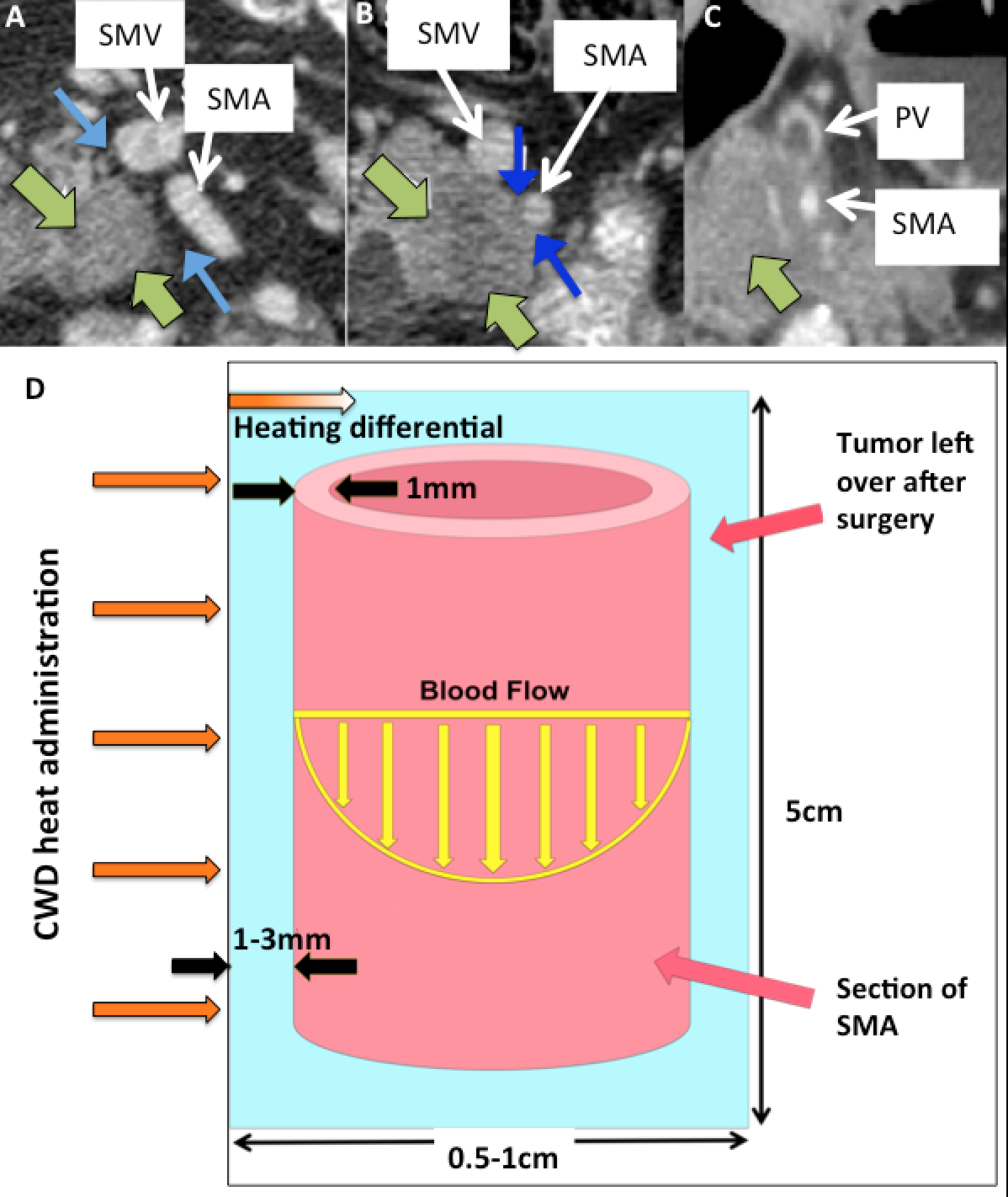


**Figure S1: Staging of patients in Resectable, borderline resectable and unresectable PDAC cohorts and schematic of artery-tumor system.** A) CT scan of resectable PDAC (large arrows); patient had no distant metastases, a clear fat plane is visible around the celiac, hepatic and mesenteric arteries (light blue arrows) and there is no abutment of superior mesenteric vein or portal vein. B) CT scan of borderline resectable PDAC; there is some tumor-SMA interaction (dark blue arrows) but <180 degrees. C) CT scan of unresectable PDAC; patient’s tumor has totally encasing the SMA (>180 degrees) (Primary tumor mass indicated by large green arrows). D) Schematic overview of CWD concept including artery, tumor and device. Surgeons can remove tumor and leave 1-3mm of positive margin. CWD heats outside of the SMA, which is the main tumor layer with endothelial tissue and smooth muscle lying underneath.


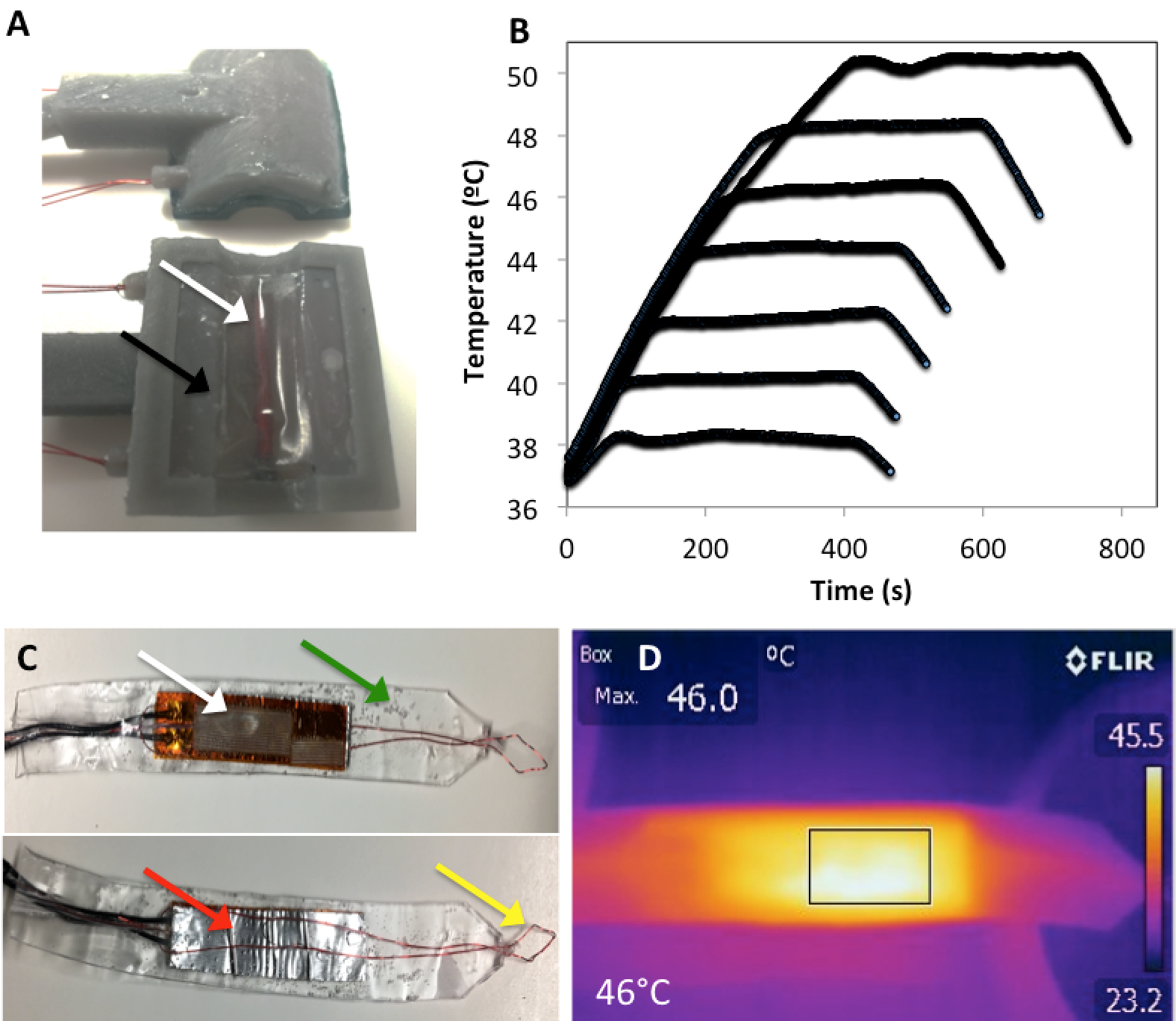


**Figure S2: Device design.** A) Images of outside and inside surface (top and bottom, respectively) of CWD designed for freely accessible positive cancer margins, for instance if a full pancreactectomy has been performed. The cylindrical design completely encloses the vessels and hyperthermia is emitted from the heating element (white arrow) before being dispersed evenly across the heating surface via the gelatin bag inside the device (black arrow). The cylindrical polymer case of the device keeps heat from non-target tissues. B) Temperature of the tuneable heating zone of the device aiming to achieve a steady state temperature for ten minutes for 37, 40, 42, 44, 46, 48 and 50°C by adjusting the electrical current running through the heating element. Measured via optic temperature probes. C) Images of inside and outside surface (top and bottom, respectively) of smaller CWD designed for positive cancer margins in tight anatomical spaces. The flexible heating element curves completely around the cancer encased artery. The insulated back of the device keeps heat from non-target tissues. D) Heating zone of device set to emit 46°C hyperthermia measured using an infrared camera.

**
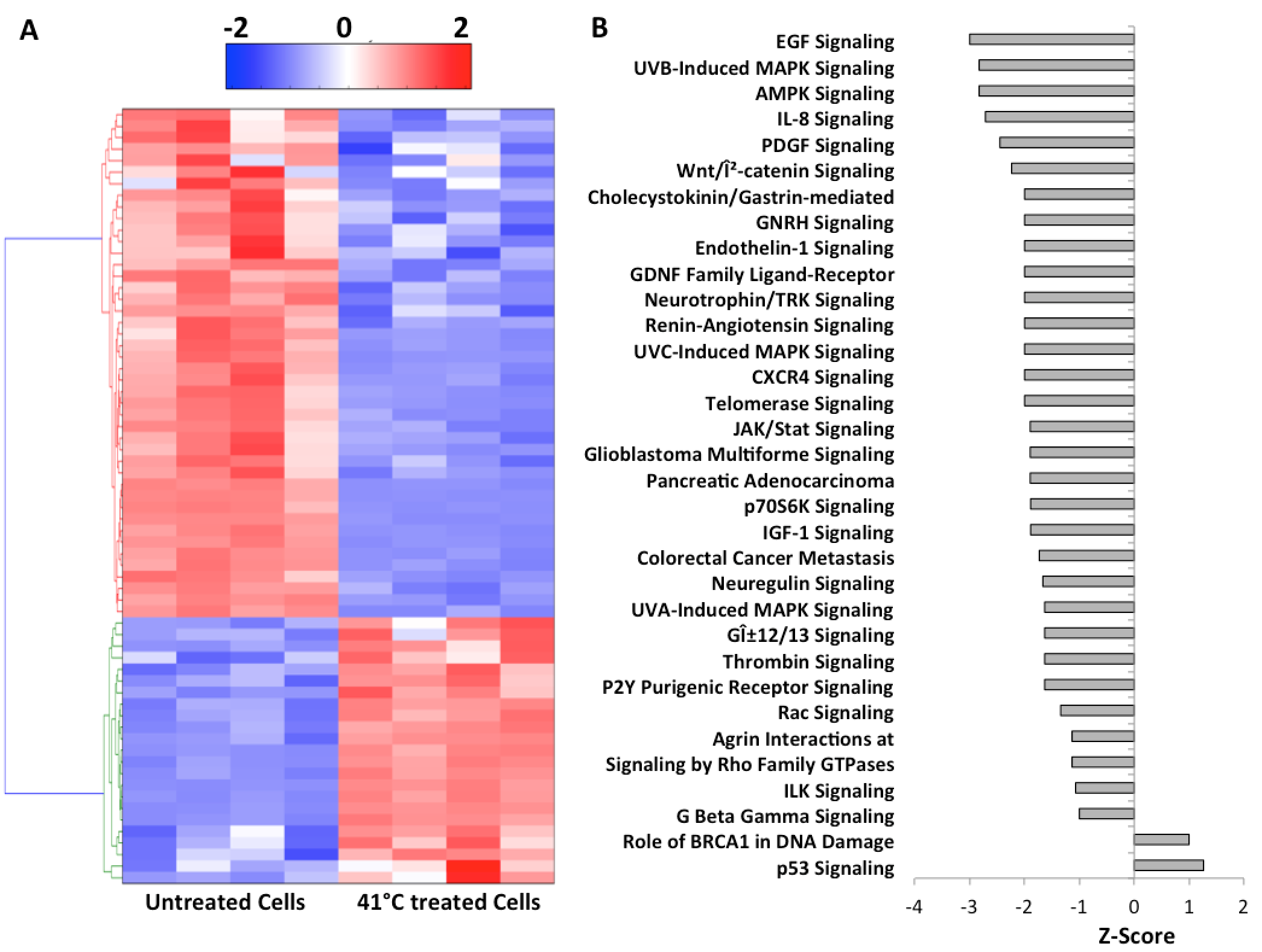
Figure S3: Proteomic analysis of PDAC cells exposed to mild hyperthermia.** A) Hierarchical clustering of RPPA data of untreated cells versus 41C^o^ (24h) treated cells (differentially expressed proteins, t test P value <0.05 and fold change > than 1.25 for up-regulation or <0.8 for down-regulation). B) Activated or suppressed canonical pathways due to RF treatment Data are displayed as the Z-score value for each pathway.

**Table S1: List of altered genes when PDAC cells were exposed to 41°C for 10 mins**

| Altered genes after 41°C hyperthermia | Relative effect | *p-value* |
| --- | --- | --- |
| Gene Symbol | (Fold change, + denotes up-  regulation and – denotes  down regulation) |
| RRM2 | +2.35 | 9.90E-09 |
| Integrin b3 | +2.06 | 5.18E-04 |
| Hexokinase II | +1.67 | 7.09E-07 |
| p-Rb | +1.34 | 2.57E-07 |
| Laminin 5 | +1.28 | 2.53E-07 |
| Slug | +1.15 | 4.53E-08 |
| Vimentin | +0.99 | 8.53E-05 |
| 14-3-3zeta, gamma, eta | +0.85 | 3.25E-03 |
| AuroraA/AIK | +0.84 | 9.66E-07 |
| DKK1 | +0.72 | 8.04E-04 |
| FGFR1 | +0.69 | 1.91E-06 |
| BRCA1 | +0.66 | 3.63E-05 |
| Integrin a5 | +0.63 | 3.88E-04 |
| GSK-3a/b | +0.54 | 2.89E-03 |
| Integrin b4 | +0.47 | 2.11E-05 |
| Caveolin-1 | +0.47 | 1.27E-05 |
| Claudin-1 | +0.41 | 2.27E-04 |
| p-Stat2 | +0.38 | 2.99E-02 |
| LC3A | +0.38 | 3.92E-03 |
| BRCA 2 | +0.38 | 2.23E-03 |
| Ki67 | +0.37 | 6.10E-03 |
| CHAF1A | +0.35 | 5.90E-05 |
| Annexin1 | +0.33 | 5.31E-03 |
| p-Aurora A | -0.33 | 2.07E-03 |
| Zeb1 | -0.34 | 1.69E-02 |
| p70S6K | -0.34 | 2.36E-02 |
| p-EGFR | -0.34 | 3.95E-03 |
| Bak | -0.35 | 6.59E-03 |
| PR | -0.37 | 1.04E-02 |
| PI3Kp110a | -0.38 | 4.58E-03 |
| p-AMPKb1 | -0.39 | 1.79E-04 |
| AMPKa | -0.39 | 2.90E-03 |
| Caspase-3 | -0.4 | 2.01E-03 |
| p-Tuberin/TSC2 | -0.4 | 8.75E-04 |
| p-ALK | -0.43 | 3.95E-04 |
| Bcl-xL | -0.43 | 3.19E-04 |
| FoxK2 | -0.44 | 1.46E-06 |
| HER2/c-ErbB2-P185 | -0.46 | 3.84E-02 |
| FSP1/S100A4 | -0.49 | 2.58E-05 |
| p-PTEN | -0.5 | 1.18E-04 |
| p-AMPKa1 | -0.5 | 3.28E-03 |
| ILK1 | -0.51 | 5.82E-04 |
| MED12-Abcam | -0.51 | 1.20E-04 |
| p-EGFR | -0.51 | 3.68E-04 |
| p-p70S6K | -0.56 | 9.82E-06 |
| p-Beta-Catenin | -0.56 | 2.74E-06 |
| PTEN | -0.59 | 9.49E-05 |
| mTOR | -0.63 | 4.88E-04 |
| Stat1 | -0.64 | 4.18E-05 |
| HER2/c-ErbB2 | -0.65 | 6.77E-04 |
| p-AMPKa | -0.67 | 1.73E-06 |
| HER3/ErbB3 | -0.67 | 1.28E-04 |
| Caspase-7 | -0.72 | 1.04E-03 |
| p-c-Fos | -0.77 | 3.75E-07 |
| p-p70S6K | -0.79 | 3.57E-05 |
| LRP6 | -0.8 | 4.49E-05 |
| p21 | -0.86 | 1.39E-05 |
| p-c-Jun | -0.89 | 7.08E-05 |
| p-mTOR | -0.94 | 5.70E-09 |
| E-Cadherin | -0.97 | 1.84E-04 |
| Bad | -1.15 | 1.75E-07 |
| p-Akt | -1.17 | 7.07E-05 |
| p-Bad | -1.75 | 1.08E-07 |
| c-Jun | -1.87 | 8.88E-08 |
| HIF-2A | -2.08 | 5.49E-04 |
| p-p44/42MAPK | -2.48 | 1.60E-07 |
| c-Fos | -2.57 | 1.08E-08 |


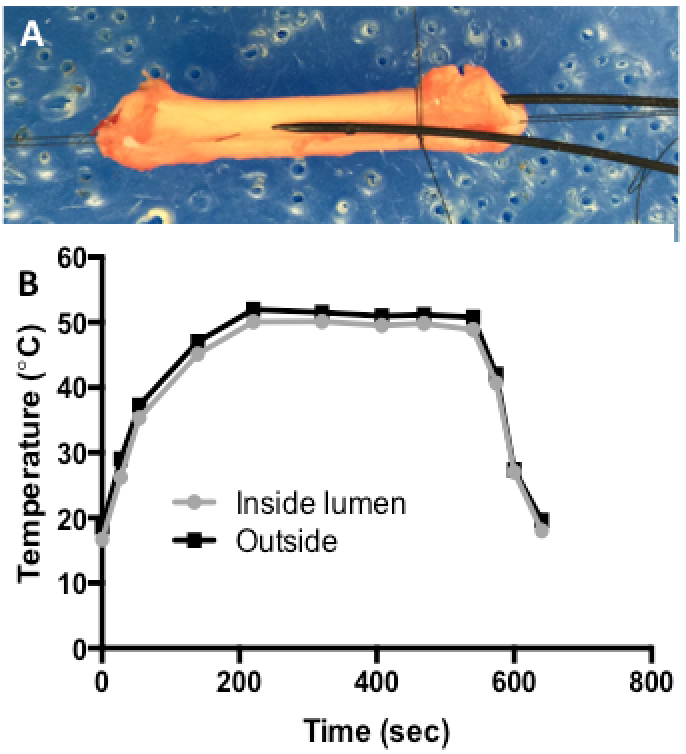


**Figure S4: Dry tissue CWD testing in *ex-vivo* Swine model.** A) Probe positioning during experimental set-up. B) Heating differential between the outside artery wall in contact with the CWD and inside arterial lumen surface.


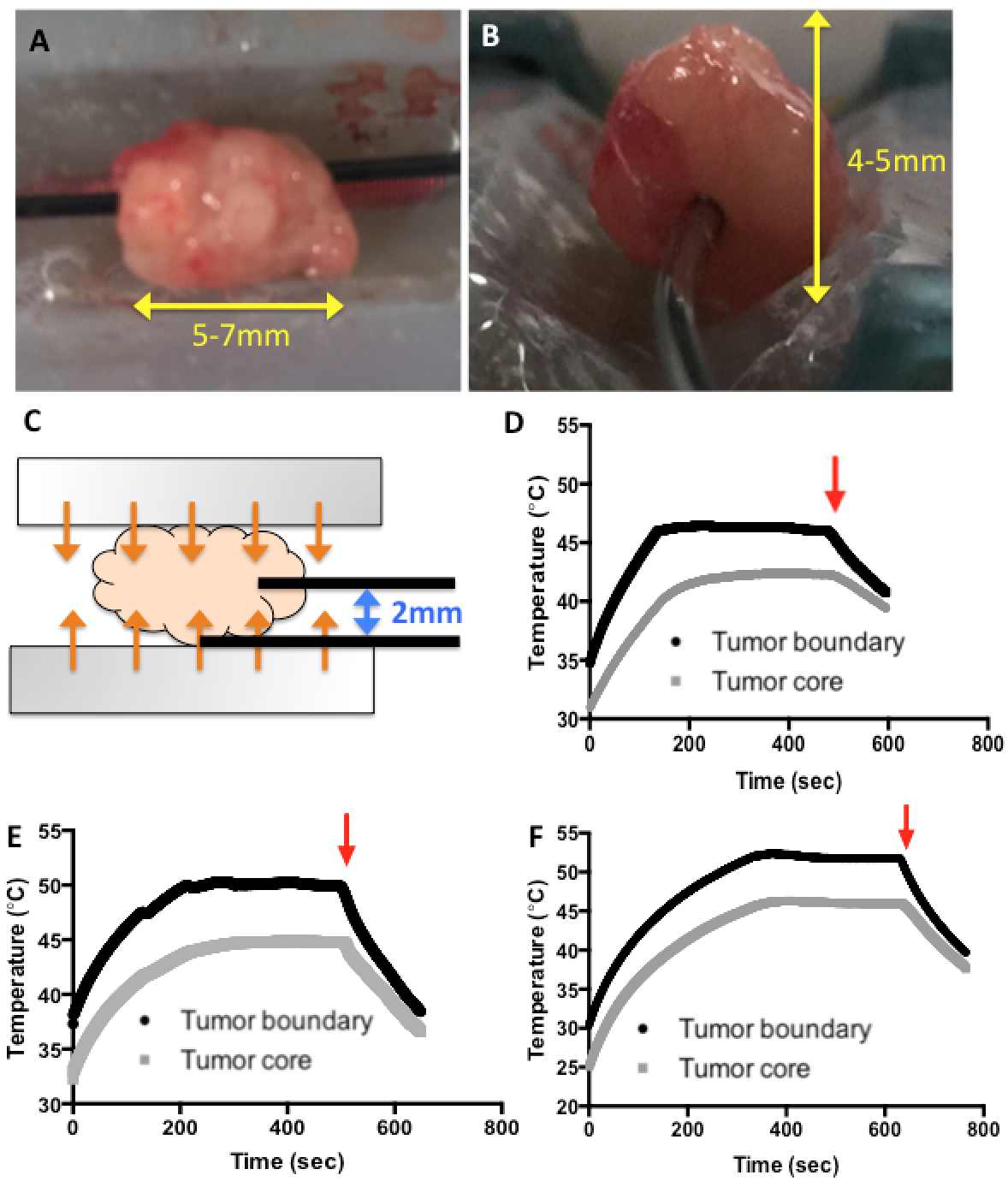


**Figure S5: Heating differential of CWD and PDAC tumor *ex-vivo*.** A) Tumor, CWD and probe orientation B) Schematic describing tumor, CWD and probe orientation during testing (P1 denotes probe 1 that is placed on tumor-CWD boundary and P2 represents probes 2 which was inserted into tumor at a 2mm distance from P1.) D, E, F) Heating differential between the tumor boundary in contact with the CWD (gray curve) and 2mm inside the PDAC tumor (black curve) for as a model for positive margin left after surgical resection., with tumor core heated to 42°C, 44°C and 46°C, respectively. Red arrow represents time point when CWD is turned off and cooling of tumor boundary and inside tumor occurs.


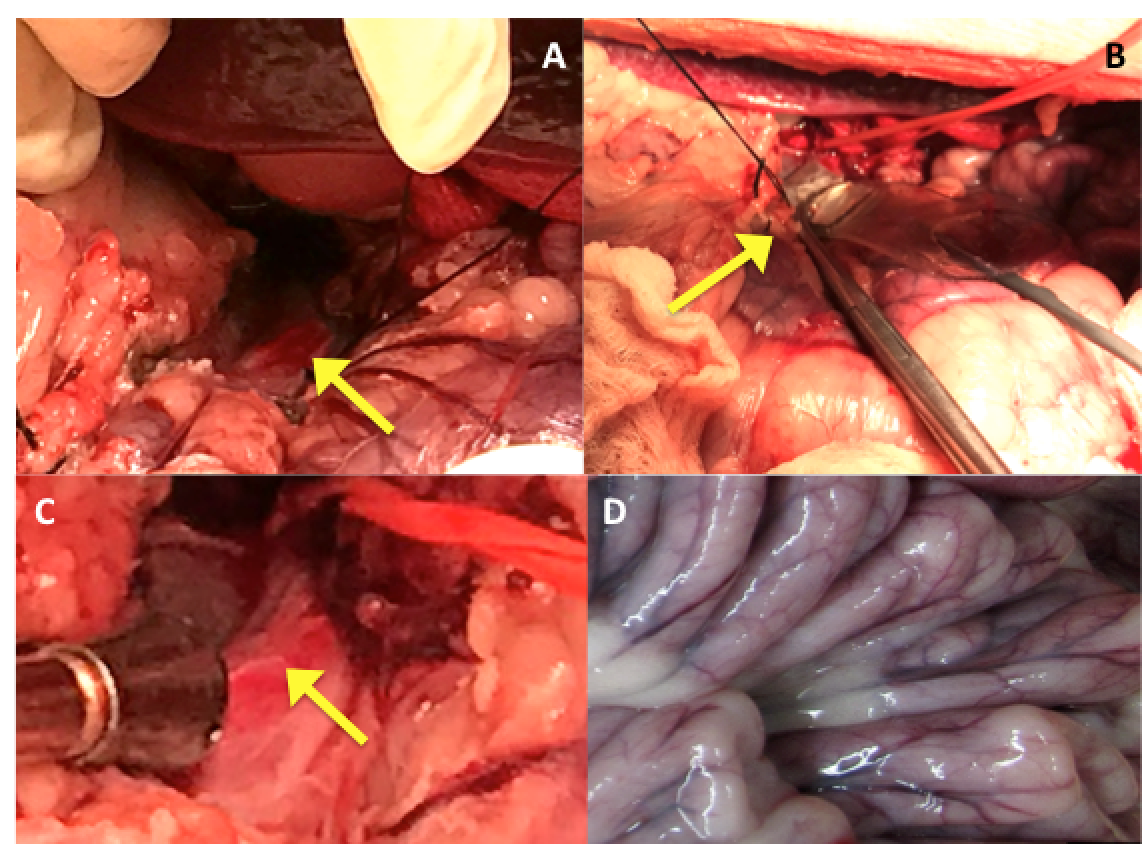


**Figure S6: Placement of the CWD, gross evaluation of tissues after surgery** (A) The exposed SMA prior to hyperthermia treatment (B) the CWD placed around SMA during hyperthermia treatment (C) the SMA post treatment (Yellow arrows indicate position of SMA) (D) the small intestine immediately after treatment.
